# Willingness to self-isolate by those with HIV

**DOI:** 10.1177/09564624221096008

**Published:** 2022-04-29

**Authors:** Haken Lane, Mark David Walker

**Affiliations:** 1 477107 Brandenburg Medical School, Neuruppin, Germany; 2 Department of the Natural and Built Environment, 7314 Sheffield Hallam University, Sheffield, UK

**Keywords:** Human immunodeficiency virus, viral disease

Throughout the COVID-19 pandemic, various behavioural measures have been advocated to reduce the risk of viral transmission.^1^ Principal amongst these has been self-isolation when infection is suspected. The Imperial College ‘COVID-19 Behavioural Tracker’^2^ collates data from online surveying of panelists on a regular basis throughout the pandemic. ‘Willingness to self-isolate if advised to do so by a healthcare professional’ (Question:i11_health) is one behaviour recorded. Respondents are also questioned whether they have any of a list of pre-existing health conditions. This provides the opportunity to examine whether compliance to self-isolation guidance varies between those with different health conditions; is there a difference between people living with HIV and other respondents? This was compared for people living with HIV (‘HIV/Aids’: d1health_1) and relatively ‘healthy’ respondents with none of a list of 13 pre-existing health conditions (d1health_99) for the U.K. Data were pooled on a monthly basis. Answers are recorded on a five-point Likert scale from ‘Very unwilling’ to ‘Very Willing’. The percentages expressing willingness (either ‘Somewhat willing’ or ‘Very willing’) was compared between the two groups.

Data from April 2020 to November 2021 were used, comprising 52 rounds of surveying. 172 people stated that they lived with HIV; 24,228 stated none of the pre-existing health conditions listed. The mean age of respondents was similar (those with HIV; 45.3, s = 13.1. No condition: 42.96, s = 15.39). Those living with HIV were predominately male (Male: 154. Female: 18). Of those stating no condition 11,556 identified as male, 12,672 as female.

Examining pooled data for the entire period, 130 of 162 people living with HIV expressed willingness to self-isolate (80.24%), while 18,039 of the 21,119 with none of the listed pre-existing conditions did (85.41%). Figure 1 show the monthly percentage willingness to self-isolate. This was lower for those living with HIV for 12 out of 19 months. Notably, willingness was lower for most of 2020, excepting April and August. For most months of 2021 willingness was comparable between the two groups. Compliance peaked initially in April 2020 at the very beginning of the pandemic (people living with HIV; 100.00; no condition; 93.88) with another peak occurring in January 2021. Differences were significant for only a single month, December 2020 (Zed proportion testing, *p* < .05).

**Figure 1.**
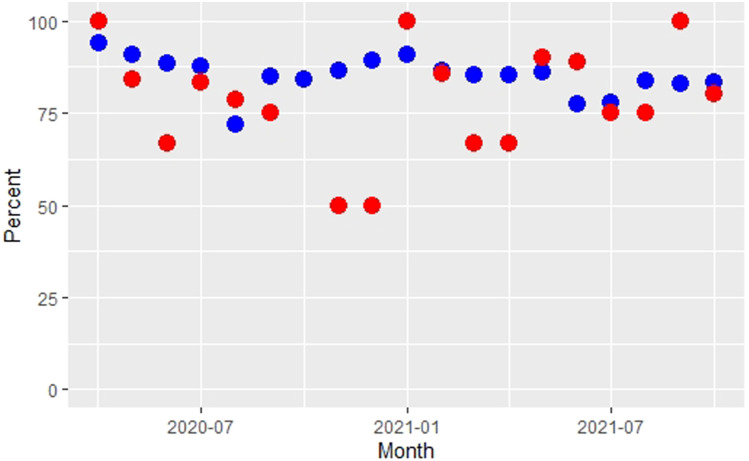
The percentage of respondents indicating willingness to self-isolate if requested to. Red: People living with HIV. Blue: No condition stated.

A limitation is obviously the small number of respondents stating that they lived with HIV; the maximum number in any 1 month being 14. Therefore, caution is required not to over interpret results. However, the apparent lower willingness to self-isolate in those living with HIV is considered of note because other studies examining the behaviour of those with a range of healthcare conditions have found a generally elevated level of adherence to COVID-19 guidance.^3,4^ Those in the ‘healthy’ group may possess conditions not recorded, thus the true level of difference is likely to be even greater than observed. Some people living with HIV have been observed to have an increased risk of adverse health complications from COVID-19,^5^ thus greater caution and compliance might be expected. However, this appears not to be the case.

The underlying factors influencing health decisions are complex, resulting from interrelating factors. These were not examined here. People living with HIV come from a diverse range of social and ethnic backgrounds meaning generalization is difficult. But those living with HIV do have a shared experience of experiencing HIV, a potentially serious condition possibly influencing their chances of survival from COVID-19. Therefore, examination as a single group, as has been done for other health conditions, was considered valid. Further study could examine compliance in those living with different stages of HIV. Further examination looking at more detail at the various factors affecting compliance in this diverse group is needed once more robust data become available.

## References

[1] Ferguson N Laydon D Nedjati Gilani G , et al. Report 9: impact of non-pharmaceutical interventions (NPIs) to reduce COVID19 mortality and healthcare demand*.* Imperial College COVID-19 Response Team. DOI: 10.25561/77482 10.25561/77482

[2] Jones SP . Imperial College London big data analytical unit and YouGov Plc. 2020, Imperial College London YouGov Covid Data Hub, v1.0, YouGov Plc, 2020. www.imperial.ac.uk/global-health-innovation/what-we-do/our-response-to-covid-19/covid-19-behaviour-tracker/

[3] Camacho-Rivera M Islam JY Vidot DC . Associations between chronic health conditions and COVID-19 preventive behaviors among a nationally representative sample of U.S. adults: an analysis of the COVID impact survey. Health Equity 2020;4(1):336–344. DOI: 10.1089/heq.2020.0031 32783017 PMC7415873

[4] Islam JY Vidot DC Camacho-Rivera M . Determinants of COVID-19 preventive behaviours among adults with chronic diseases in the USA: an analysis of the nationally representative COVID-19 impact survey. BMJ Open 2021;11(2):e044600. DOI: 10.1136/bmjopen-2020-044600 10.1136/bmjopen-2020-044600 PMC7874902 33563624

[5] Ssentongo P Heilbrunn ES Ssentongo AE , et al. Epidemiology and outcomes of COVID-19 in HIV-infected individuals: a systematic review and meta-analysis. Sci Rep. 2021;11(1):1–2. DOI: 10.1038/s41598-021-85359-3 33737527 PMC7973415

